# Prolonged neoadjuvant chemotherapy without radiation versus total neoadjuvant therapy for locally advanced rectal cancer: A propensity score matched study

**DOI:** 10.3389/fonc.2022.953790

**Published:** 2022-09-16

**Authors:** Xuan Zhao, Peiyi Han, Luyang Zhang, Junjun Ma, Feng Dong, Lu Zang, Zirui He, Minhua Zheng

**Affiliations:** ^1^ Department of General Surgery, Ruijin Hospital, Shanghai Jiao Tong University School of Medicine, Shanghai, China; ^2^ Shanghai Minimally Invasive Surgery Center, Shanghai, China

**Keywords:** chemoradiotherapy, rectal cancer, neoadjuvant chemotherapy, recurrence, propensity score

## Abstract

**Background:**

Although neoadjvuant chemoradiotherapy (CRT) improves the local control rate of locally advanced rectal cancer (LARC), it fails to significantly improve disease-free survival (DFS) and overall survival (OS). We explored the efficacy of prolonged neoadjuvant chemotherapy (pNCT) without radiation and compared this schema with total neoadjuvant therapy (TNT).

**Material and methods:**

Patients diagnosed with LARC and received TNT (4 cycles of induction CapeOX/FOLFOX followed with CRT) or pNCT (6~8 cycles of CapeOX/FOLFOX) between June 2016 and October 2021 were retrospective analyzed. All patients underwent total mesorectal excision (TME). A 1:1 propensity score match was performed to adjust baseline potential confounders. The tumor response, toxicity, recurrence-free survival (RFS) and OS were observed.

**Results:**

A total of 184 patients with 92 patients in each group were finally enrolled. The median follow-up time was 35 months. TNT showed better pathological complete response (pCR) rate (25.0% vs 16.3%) and objective regression rate (73.9% vs 59.8%) than pNCT. TNT and pNCT produce similar 3-year RFS and OS rates in patients with mid-to-upper rectal cancer. TNT was associated with improved tumor responsiveness in all patients and improved 3-year RFS rates in those with low rectal cancer.

**Conclusion:**

pNCT is an option for patients with mid-to-upper rectal cancer, but radiation is still necessary for low rectal cancer. To determine optimal schema for neoadjuvant therapy and patient selection, additional randomized controlled studies are needed.

## 1 Introduction

Neoadjuvant chemoradiotherapy (CRT) followed by total mesorectal excision (TME) and adjuvant systemic chemotherapy comprise the general paradigm of locally aggressive rectal cancer (LARC) treatment. Although the treatment strategy improves the local control of disease (1–4), it fails to significantly improve disease-free survival (DFS) and overall survival (OS) (5). Distant recurrence remains the leading cause of death for patients and is inadequately controlled by the current treatment mode (6, 7).

Recent evidence suggests that neoadjuvant chemotherapy may be used for controlling distant recurrence. Combined chemotherapy and chemoradiation, referred to as total neoadjuvant therapy (TNT), is used worldwide, and several high-quality trials showed that TNT improves oncological outcomes in two aspects (8–11). First, short-term recurrence rates decreased in those who underwent TNT, especially at 3-year follow up. Second, pathological complete response (pCR) rates increased after treatment with TNT, with the therapy nearly doubling pCR rates compared with CRT.

In recent years, use of neoadjuvant chemotherapy alone was reported to result in promising survival outcomes (12, 13). The FORWARC (13) study showed that 3-year DFS rates in those undergoing mFOLFOX6 with and without routine radiation did not significantly differ, and the elimination of radiation was unlikely to increase local recurrence risk after R0/1 resection. Especially in low-risk patients whose response to chemotherapy was good, the need for radiotherapy remains unclear. Findings of the trials have the potential to update clinical practice guidelines regarding the use of radiotherapy. To date, the optimal use of neoadjuvant chemotherapy and its target patient population remains controversial. The ideal way to maintain a balance between the benefits of TNT and overtreatment is of particular importance, and is likely to be debated well into the future.

In this study, we retrospectively reviewed records of 257 patients who were diagnosed with resectable LARC and treated with either TNT or prolonged neoadjuvant chemotherapy (pNCT) without radiation. We aimed to assess the safety, efficacy, and survival outcomes of pNCT versus TNT in patients with baseline resectable LARC.

## 2 Material and methods

### 2.1 Study population

This retrospective study utilized the data of consecutive patients who underwent TNT/pNCT followed with radical surgery for rectal cancer between June 2016 and October 2021 at Ruijin Hospital, Shanghai, China. Patients were randomly assigned to receive TNT or pNCT and were staged using preoperative imaging, including enhanced magnetic resonance imaging (MRI) and computed tomography (CT). Other clinical data were obtained from the patients’ medical history at Ruijin Hospital.

Patients meeting the following eligibility criteria were included: (I) aged between 18 and 80 years; (II) diagnosis of rectal adenocarcinoma *via* colonoscopy and a pathological examination (the lower tumor edge within 12 cm of the anal verge); (III) underwent TNT or pNCT, and (IV) postoperative pathological results showing R0 resection. Exclusion criteria were as follows: (I) the presence of concomitant malignant disease, (II) history of malignant disease, (III) failure to complete planned cycles of neoadjuvant or adjuvant treatment, and (IV) unresectable tumors or difficult to get R0 resection after neoadjuvant therapy.

### 2.2 Treatment

#### 2.2.1 Neoadjuvant therapy and adjuvant therapy

All patients received oxaliplatin- and fluorouracil-based neoadjuvant chemotherapy (mFOLFOX6 or CapeOX). The mFOLFOX6 regimen consisted of an intravenous infusion of oxaliplatin (85 mg/ m^2^) followed by leucovorin (400 mg/m^2^), an intravenous bolus of 5-FU (400 mg/m^2^), and a continuous intravenous infusion of 5-FU (2,400 mg/m^2^) for 2 days. The CapeOX regimen consisted of intravenous infusion of oxaliplatin (130 mg/m^2^). Capecitabine (1,000 mg/m^2^) was orally administered twice daily for 14 days. The pNCT group received six to eight cycles of chemotherapy. After surgery, patients diagnosed with pathological stage III or high-risk stage II rectal adenocarcinoma received adjuvant therapy similar to the preoperative treatment for an additional four cycles. The high-risk factors included: CRM<1mm, ypN2/N1c, poor mesorectal quality, poor tumor differentiation.

#### 2.2.2 Synchronous chemoradiotherapy

The TNT group received CRT after four cycles of induction chemotherapy. Patients received 50 Gy radiation throughout 5 weeks (2 Gy five times per week). During radiotherapy, continuous oral capecitabine was administered twice daily, on days 1–14 and 22–35. This procedure was performed by specific radiation oncologists.

### 2.3 Surgery and pathological examination

CT and enhanced rectal MRI imaging were repeated after preoperative treatment. Surgery was performed if the tumor was considered resectable. In the pNCT group, the median interval between the last treatment and surgery was 2 week (range, 2-3 weeks). In the TNT group, the median interval between CRT and surgery was 6 weeks (range, 4-6 weeks), with one cycle of chemotherapy during the interval. All included patients underwent laparoscopic TME with R0 resection. The surgical specimens were examined by pathologists from the Department of Pathology. Pathological features such as ypT stage, ypN stage, tumor differentiation, and tumor response were determined *via* routine methods, and a mismatch repair status (MMR) test was performed, if necessary. The radiological response to neoadjuvant therapy was evaluated based on response evaluation criteria for solid tumors (RECIST v1.1).

### 2.4 Post-treatment surveillance

All patients were followed-up every 3 months for the first year after surgery, and every 6 months for the next 4 years. Enhanced CT scans (chest, abdomen, and pelvis), serum tests for tumor markers (CEA, CA19-9, CA125, and CA242), and colonoscopies were performed every six months. Survival outcomes and the recurrence status of patients were also noted. Recurrence-free survival (RFS) was defined as the time between the end of treatment and date of recurrence. OS was defined as the time from surgery to the date of all-cause death.

### 2.5 Statistical analysis

Statistical analyses were performed using IBM SPSS Statistics version 26 (IBM Corporation, Armonk, NY, USA) or R version 4.1.3. Propensity scores matching (PSM) at a ratio of 1:1 was completed using SPSS. The chi-square test was used to compare categorical variables, and continuous variables were analyzed using the Student’s t-test. Kaplan–Meier curves were used to analyze survival and recurrence. A Cox regression model was used to calculate hazard ratios of OS and RFS. Survival curves and forest plots were constructed using the R packages *survminer*, *forestmodel* and *forestplot*. P-values were two-sided, and P < 0.05 was considered statistically significant.

## 3 Results

### 3.1 Patient characteristics

**Figure 1:**
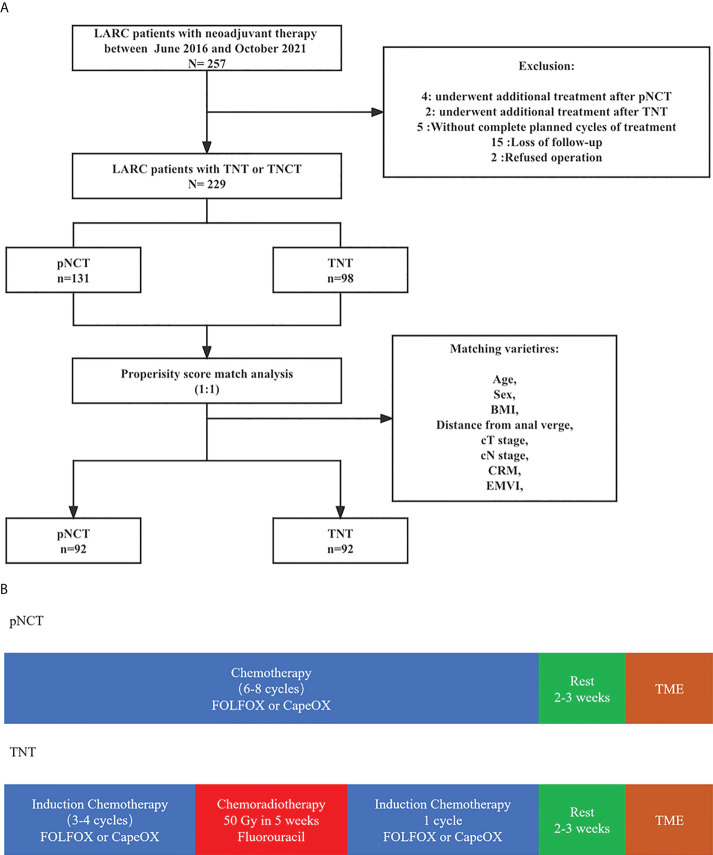
The diagram of total study **(A)** Neoadjuvant treatment; **(B)** Study population.The diagram of total study **(A)** Neoadjuvant treatment; **(B)** Study population. pNCT prolonged neoadjuvant chemotherapy; TNT, total neoadjuvant therapy; BMI, Body mass index; CRM, circumference resection margin; EMVI, extramural vascular invasion; TME, total mesorectal excision.

A total of 257 patients with LARC who underwent TNT or pNCT from April 2016 to August 2021 were enrolled in this study (**Figure 1**). A total of 229 patients were included after screening, with 131 and 98 being treated with pNCT and TNT, respectively. Among the excluded patients, 4 and 2 patients in the pNCT and TNT groups (2.96% vs 1.72%), respectively, were excluded due to unsatisfied tumor response, and underwent additional treatment later (**Supplementary Table1**). Finally, 92 patients of each group were studied after a 1:1 PSM. Baseline clinical characteristics of patients before and after PSM are shown in **Table 1**. Overall, 176 of 184 patients (96%) had cT3 and cT4 tumors, and 174 of 184 patients (95%) had clinically involved lymph nodes. The mean distance to the anal verge was 5.95 cm, and 71 of 184 patients (39%) had low rectal cancer (within 5 cm from the anal verge).

**Table 1:** Baseline clinical characteristics of patients before and after propensity scoring matching.

Characteristic		Before matching			After matching		
		pNCTN=131	TNTn=98	P	pNCTN=92	TNTN=92	P
Sex (%)	Male	103 (78.6)	76 (77.6)	0.974	74 (80.4)	70 (76.1)	0.592
	Female	28 (21.4)	22 (22.4)		18 (19.6)	22 (23.9)	
Age, years (SD)		60.91 (9.23)	60.90 (8.68)	0.993	61.23 (8.64)	60.60 (8.61)	0.621
BMI (SD)		22.93 (3.32)	22.92 (2.53)	0.993	22.87 (3.08)	22.93 (2.56)	0.871
Distance from anal verge, cm (SD)		6.34 (2.32)	6.02 (1.99)	0.282	5.95 (1.97)	6.01 (2.01)	0.853
Pretreatment CEA level, ng/ml (SD)		7.31 (13.69)	4.52 (7.76)	0.071	4.42 (5.63)	3.61 (4.76)	0.293
cT (%)	cT1	0 (0.0)	2 (2.0)	0.208	0 (0.0)	2 (2.2)	0.275
	cT2	4 (3.1)	4 (4.1)		3 (3.3)	3 (3.3)	
	cT3	65 (49.6)	39 (39.8)		48 (52.2)	38 (41.3)	
	cT4	62 (47.3)	53 (54.1)		41 (44.6)	49 (53.3)	
cN (%)	cN0	10 (7.6)	4 (4.1)	0.516	6 (6.5)	4 (4.3)	0.718
	cN1	49 (37.4)	40 (40.8)		34 (37.0)	38 (41.3)	
	cN2	72 (55.0)	54 (55.1)		52 (56.5)	50 (54.3)	
CRM (%)	Negative	52 (39.7)	32 (32.7)	0.339	29 (31.5)	31 (33.7)	0.875
	Positive	79 (60.3)	66 (67.3)		63 (68.5)	61 (66.3)	
EMVI (%)	Negative	53 (40.5)	37 (37.8)	0.781	35 (38.0)	35 (38.0)	1
	Positive	78 (59.5)	61 (62.2)		57 (62.0)	57 (62.0)	
Median Follow-up Time months (min-max)					34.5 (9-61)	36 (5-64)	

pNCT, prolonged neoadjuvant chemotherapy; TNT, total neoadjuvant therapy; BMI, Body mass index; CRM, circumference resection margin; EMVI, extramural vascular invasion.

### 3.2 Pathology staging and response to chemotherapy

All patients underwent laparoscopic TME and a pathological examination to evaluate their responsiveness to treatment preoperatively. Similar ypT and ypN stages were observed after both TNT and pNCT. TRG findings revealed that the TNT group had a higher rate of TRG0 and TRG1 than the pNCT group; however, the difference was not statistically significant (p = 0.095, **Table 2**). Patients in the TNT group had higher pathological complete response (pCR; 25.0% and 16.3%, respectively) and objective regression rates (73.9% and 59.8%, respectively) than those of the pNCT group. TNT and pNCT groups showed promising disease control rates (93.5% and 95.7%, respectively). As for toxicity, no patient died of chemotherapy-related adverse events. Grade 3/4 adverse events in preoperative chemotherapy and postoperative hospitalization were rare in both groups (**Table 3**). The most common severe adverse event was leukopenia, which was similar between the two groups (4 in pNCT vs. 3 in TNT, p=0.702).

**Table 2:** Pathological result of tumor response to neoadjuvant therapy.

Characteristics		OverallN=184	pNCTN=92	TNTN=92	P value
ypT (%)	ypT0	40 (21.7)	16 (17.4)	24 (26.1)	0.512
	ypT1	1 (0.5)	0 (0.0)	1 (1.1)	
	ypT2	24 (13.0)	12 (13.0)	12 (13.0)	
	ypT3	82 (44.6)	44 (47.8)	38 (41.3)	
	ypT4	37 (20.1)	20 (21.7)	17 (18.5)	
ypN (%)	ypN0	106 (57.6)	50 (54.3)	56 (60.9)	0.112
	ypN1	43 (23.4)	19 (20.7)	24 (26.1)	
	ypN2	35 (19.0)	23 (25.0)	12 (13.0)	
Differentiation (%)	No tumor*	42 (22.8)	16 (17.4)	26 (28.3)	0.02
	Poor	22 (12.0)	15 (16.3)	7 (7.6)	
	Moderate	76 (41.3)	33 (35.9)	43 (46.7)	
	Well	44 (23.9)	28 (30.4)	16 (17.4)	
TRG (%)	0	39 (21.2)	15 (16.3)	24 (26.1)	0.095
	1	13 (7.1)	4 (4.3)	9 (9.8)	
	2	100 (54.3)	53 (57.6)	47 (51.1)	
	3	32 (17.4)	20 (21.7)	12 (13.0)	
Response (%)	CR*	38 (20.7)	15 (16.3)	23 (25.0)	0.408
	PR	76 (41.3)	40 (43.5)	36 (39.1)	
	SD	60 (32.6)	33 (35.9)	27 (29.3)	
	PD	10 (5.4)	4 (4.3)	6 (6.5)	
ORR (%)	SD+PD	70 (38.0)	37 (40.2)	24 (26.1)	0.06
	CR+PR	114 (62.0)	55 (59.8)	68 (73.9)	
DCR (%)	PD	10 (5.4)	4 (4.3)	6 (6.5)	0.745
	CR+PR+SD	174 (94.6)	88 (95.7)	86 (93.5)	
pCR (%)	PR+SD+PD	146 (79.3)	77 (83.7)	69 (75.0)	0.202
	CR	38 (20.7)	15 (16.3)	23 (25.0)	

pNCT, prolonged neoadjuvant chemotherapy; TNT, total neoadjuvant therapy; ypT, Pathological T stage after neoadjuvant therapy; ypN, Pathological N stage after neoadjuvant therapy; TRG, tumor regression grade; CR, complete response; PR, partial response; SD, stable disease; PD, progressive disease; ORR, Objective regression rate; DCR, Disease control rate; pCR, Pathological complete regression.

*The number of “No tumor” patients were more than “CR” patients because four patients had complete tumor regression but still had positive lymph nodes.

**Table 3:** Comparison of toxicity and adverse event.

Adverse Event	pNCTn=92	TNTn=92	P
Chemotherapy-related adverse event (%)			
Death	0	0	
Leukopenia (Grade 3, 4^†^)	4 (4.35)	3 (3.26)	0.702
Anemia (Grade 3, 4^†^)	0	1 (1.09)	0.319
Thrombocytopenia (Grade 3, 4^†^)	1 (1.09)	0	0.319
Diarrhea (Grade 3, 4^†^)	2 (2.18)	1 (1.09)	0.563
Postoperative complications (%)			
Death	0	0	
Bleeding (Grade 3, 4^#^)	1 (1.09)	2 (2.18)	0.563
Anastomosis leakage (Grade B, C^*^)	5 (5.43)	7 (7.61)	0.234
Wound infection (Grade 3, 4^#^)	0	1 (1.09)	0.319

^†^Common Terminology Criteria for Adverse Events (CTCAE).

^#^Clavien-Dindo classification.

^*^Classification of International Study Group of Rectal Cancer (ISREC).

### 3.3 Surgical outcomes and survival

The permanent diversion rate was 20.65% (19/92) and 27.17% (25/92) in the pNCT and TNT group, and the temporary stoma rate was 29.35% (27/92) and 26.09% (24/92), respectively. Anastomosis bleeding and leakage were the most common short-term complications. Five patients in pNCT group and seven patients in TNT group suffered from grade 3/4 anastomosis leakage and showed no significant difference between two groups (5.43 vs. 7.61, p=0.234).

**Figure 2:**
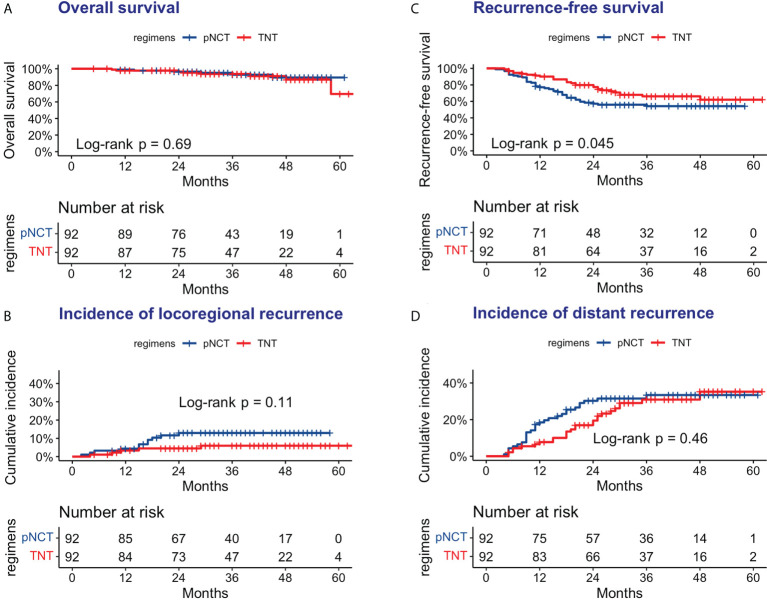
Oncologic outcomes of pNCT group and TNT group **(A)** Overall survival **(B)** Disease-free survival; **(C)** Cumulative incidence of locoregional recurrence; **(D)** Cumulative incidence of distant metastases. pNCT prolonged neoadjuvant chemotherapy; TNT total neoadjuvant therapy.

The median follow-up period was 35 months (5–64 months), with that for the pNCT and TNT groups being 34.5 months (9–61 months) and 36 months (5–64 months), respectively. As shown in **Figure 2**, 6 of 92 patients of the pNCT group (6.5%) and 8 (8.7%) of the TNT group died. Recurrence was reported in 40 (43.5%) and 29 (31.5%) patients of the pNCT and TNT groups, respectively (**Table 4**). Among these patients, local recurrence events were reported in 11 (12.0%) and 5 (5.4%) patients of the pNCT and TNT groups, respectively, and distant metastasis was reported in 30 (33.6%) and 26 (28.3%) patients.

**Table 4:** Comparison of outcomes of patients.

Relapse Type	pNCTn=92	TNTn=92	P
Death (%)	6 (6.5)	8 (8.7)	0.781
Total (%)	40 (43.5)	29 (31.5)	0.128
Locally recurrence (%)	11 (12.0)	5 (5.4)	0.191
Distant metastasis (%)	30 (33.6)	26 (28.3)	0.747
Liver (%)	21 (22.8)	17 (18.5)	0.585
Lung (%)	4 (4.3)	4 (4.3)	1.000
Liver+Lung(%)	4 (4.3)	3 (3.3)	1.000
Other (%)	1 (1.1)	2 (2.2)	1.000

pNCT, prolonged neoadjuvant chemotherapy; TNT, total neoadjuvant therapy.

**Figure 3:**
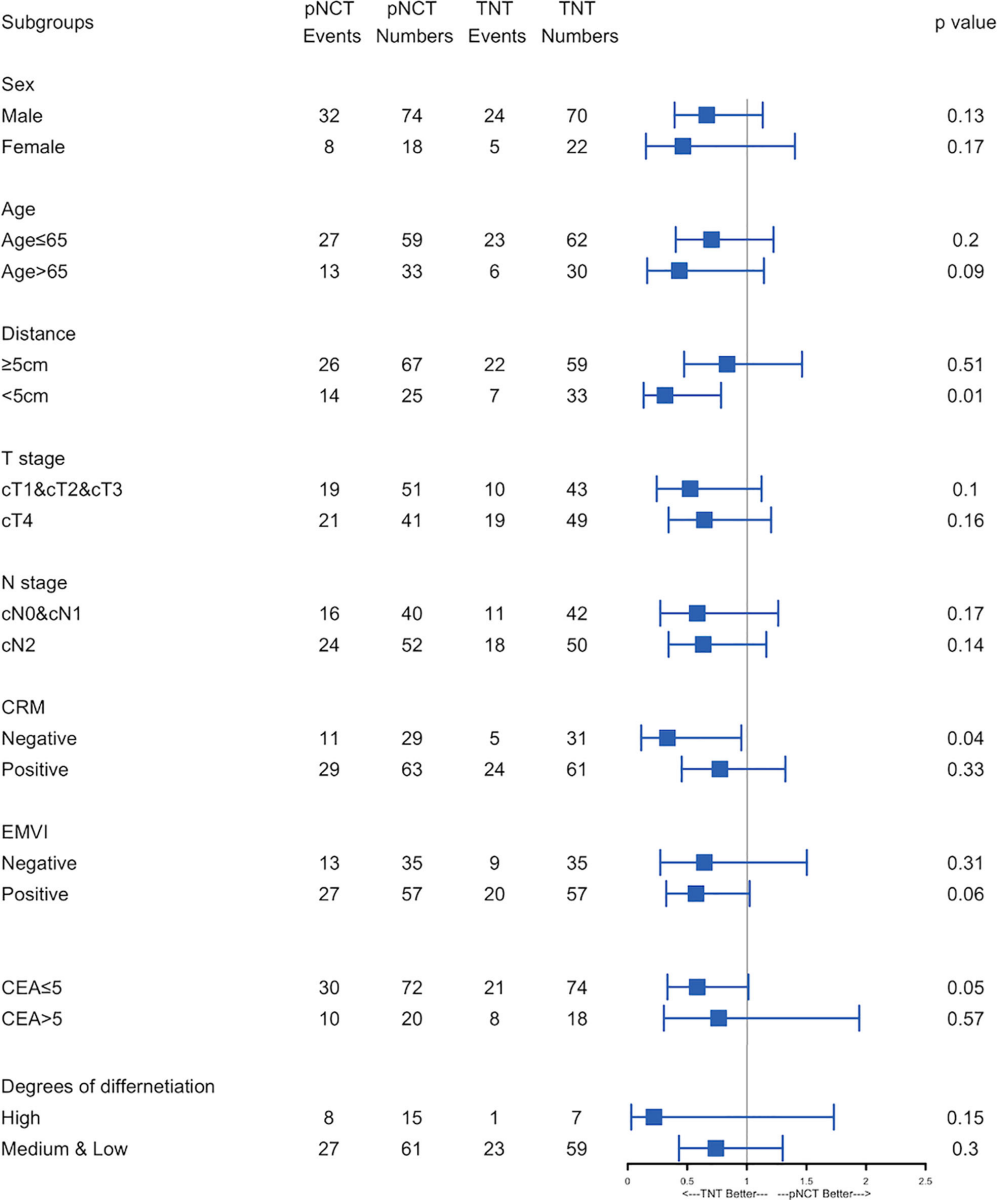
Forest plot of recurrence-free survival.Forest plot of recurrence-free survival. Subgroups analyses of recurrence-free survival was performed. pNCT, prolonged neoadjuvant chemotherapy; TNT, total neoadjuvant therapy; CRM, circumference resection margin; EMVI, extramural vascular invasion; ypT, Pathological T stage after neoadjuvant therapy; ypN, Pathological N stage after neoadjuvant therapy; CR, complete response; PR, partial response; SD, stable disease; PD, progressive disease.

**Figure 4:**
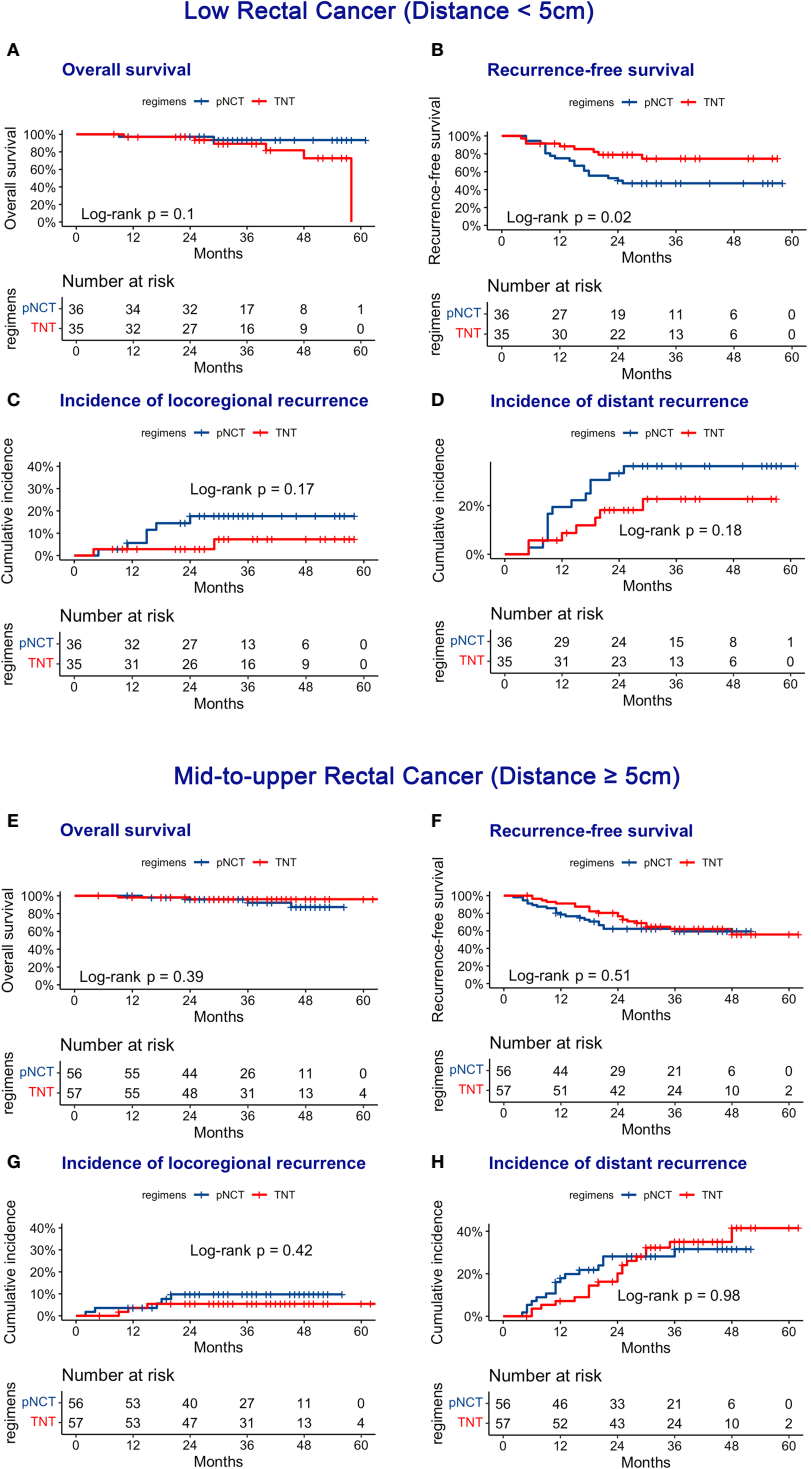
Oncologic Outcomes of pNCT and TNT in subgroup. Subgroup was divided according to the distance between tumor and anal verge. **(A)** Overall survival **(B)** Disease-free survival; **(C)** cumulative incidence of locoregional recurrence; **(D)** cumulative incidence of distant metastases in low group (distance <5 cm). **E** Overall survival **(F)** Disease-free survival; **(G)** cumulative incidence of locoregional recurrence; **(H)** cumulative incidence of distant metastases in mid-to-upper group (distance ≥5 cm).

Subgroup analysis of survival outcomes showed that low rectal cancer (within 5 cm of the anal verge) was a strong indicator of TNT (P <0.01, **Figure 3**), while other factors assessed failed to show statistical significance. We further investigated survival outcomes of patients with and without low rectal cancer. Results showed that for patients with low rectal cancer, better RFS was associated with the use of TNT; however, this result was not observed in patients with mid-to-upper rectal cancer (**Figure 4**). In univariable and multivariable analysis, effects of these factors on RFS and OS were assessed (**Table 5**). Worsened RFS was associated with pNCT, cT4, ypT4, ypN2, and unfavorable tumor response, while ypT4 (HR, 2.00; CI,1.20–3.50) and ypN2 (HR,2.20; CI, 1.30–3.60) were independent risk factors.

**Table 5:** Univariable and multivariable analysis of the effects of prognostic factors on recurrence-free survival and overall survival.

Chatacteristic	RFS				OS	
	uni-HR (HR.95L-HR.95H)	pvalue	Multi-HR (HR.95L-HR.95H)	pvalue	HR (HR.95L-HR.95H)	pvalue
Regimens (pNCT vs TNT)	0.66 (0.38-0.99)	0.047	0.67 (0.41-1.10)	0.110	0.66 (0.43-3.60)	0.689
Sex (Female vs Male)	1.20 (0.63-2.12)	0.634	——	——	1.20 (0.45-26.14)	0.237
Age (Age<=65 vs Age>65)	0.65 (0.40-1.16)	0.155	——	——	0.65 (0.34-3.01)	0.991
cT_Stage (cT1&cT2&cT3 vs cT4)	1.30 (1.02-2.66)	0.04	1.30 (0.79-2.20)	0.300	1.30 (1.11-11.99)	0.033
cN_Stage (cN0 vs cN1&cN2)	1.00 (0.52-27.22)	0.187	——	——	1.00 (0-Inf)	0.998
MMR (unknown&pMMR vs dMMR)	1.50 (0.44-1.15)	0.165	——	——	1.50 (0.44-3.62)	0.665
CEA (CEA ≤ 5 vsCEA>5)	0.86 (0.87-2.56)	0.143	——	——	0.86 (0.04-2.47)	0.277
CRM (negative vs positive)	2.00 (0.96-2.95)	0.067	——	——	2.00 (0.50-6.49)	0.365
EMVI (negative vs positive)	2.20 (0.81-2.23)	0.252	——	——	2.20 (0.22-1.83)	0.405
ypT_Stage (ypT1&ypT2&ypT3 vs ypT4)	0.77 (1.53-4.23)	<0.001	2.00 (1.20-3.50)	0.012	0.77 (0.61-6.20)	0.265
ypN_Stage (ypN0 vs ypN1&ypN2)	1.20 (1.57-4.13)	<0.001	2.20 (1.30-3.60)	0.003	1.20 (1.00-8.98)	0.05
Response (CR&PR vs SD&PD)	1.30 (1.21-3.11)	0.006	1.40 (0.86-2.30)	0.180	1.30 (0.43-3.64)	0.68
Distance (≥5cm vs <5cm)	1.20 (0.60-1.67)	0.997	——	——	1.20 (0.75-6.12)	0.156

pNCT, prolonged neoadjuvant chemotherapy; TNT, total neoadjuvant therapy; CRM, circumference resection margin; EMVI, extramural vascular invasion; MMR, mismatch repair; ypT, Pathological T stage after neoadjuvant therapy; ypN, Pathological N stage after neoadjuvant therapy; CR, complete response; PR, partial response; SD, stable disease; PD, progressive disease.

## 4 Discussion

Recently, TNT has been widely used to treat patients with LARC, which results in promising survival outcomes. One of the advantages of TNT is that it allows for the early use of systemic chemotherapy, which may improve the efficiency by which micro-metastases at early stages of tumor development are targeted (14, 15). However, with increasing focus on neoadjuvant chemotherapy, the value of preoperative radiation, especially in patients with initially resectable tumors that undergo high-quality TME surgery, is under question. Recently, several randomized studies have reported non-inferior survival outcomes in those receiving neoadjuvant chemotherapy without radiation (12, 13, 16, 17), which has prompted a reexamination of the significance of preoperative radiation.

In our study, we compared prolonged neoadjuvant chemotherapy without radiation to TNT composed of induction CapeOX/FOLFOX and CRT. All patients underwent R0 resection, as confirmed *via* a pathological examination. Results showed that the pCR and 3-year RFS rates of the TNT group were longer than those of the pNCT group; however, TNT failed to improve survival in those with middle and high rectal cancer. This result showed that TNT may related with improved tumor responsiveness and promising survival outcomes; however, preoperative radiation might not be necessary for initially resectable mid-to-upper rectal cancer.

Previous studies have reported that although neoadjuvant radiotherapy may improve the pCR rate, it fails to improve the prognosis. Moreover, radiation may damage normal tissue adjacent to the tumor, a process that may be related to several complications, including radiation-induced rectal injury, anastomosis leakage, sexual dysfunction, and bowel dysfunction (18, 19). Recently, several prospective clinical studies have assessed the effectiveness of radiotherapy-free regimens. The FOWARC trial compared the following treatment regimens: neoadjuvant mFOLFOX6 alone, fluorouracil plus radiotherapy, and mFOLFOX6 plus radiotherapy. Results showed that outcomes of those undergoing mFOLFOX6 with or without radiation and fluorouracil with radiation did not significantly differ (13). Deng et al. (16) assessed outcomes in those given neoadjuvant CapeOX alone, reporting that promising tumor response rates were observed. Zhang et al. (12) administered neoadjuvant chemotherapy alone with triplet regimens of mFOLFOXIRI, which also produced similar oncologic outcomes. On the other hand, it is still not clear whether all patients with LARC need chemoradiotherapy. The QuickSilver trial studied MRI-predicted good prognosis rectal patients, and suggested that CRT might not be necessary for stage II and III rectal cancer (20). The prospective multicenter OCUM trial compared surgery alone versus neoadjuvant CRT, and showed a better DFS and OS for high-risk patients (CRM+ or lower third rectal cancer) (21).These studies indicated that the application of radiotherapy needs more accurate selection of patients.

Our study shows that chemotherapy alone produces similar RFS and OS in patients with middle and high rectal cancer; however, chemoradiotherapy remained necessary in those undergoing low rectal cancer treatment. The following three explanations for this result are possible. First, the lower pelvic cavity is narrower than the upper pelvic cavity. Performing lower rectal cancer radical resection of the complete mesorectum with a sufficiently resection margin is difficult. In these cases, preoperative radiation could improve tumor downstaging more than chemotherapy alone, thereby limiting the risk of residual tumors. Second, the tumor location is a prognostic factor for assessing the treatment efficacy (22). Patients with lower rectal cancer may benefit from neoadjuvant radiation more than those undergoing upper rectal cancer therapy (23). Third, the increased rate of postoperative complications in low rectal cancer may delay treatment after surgery.

Although several clinical trials have focused on assessing the use of neoadjuvant treatment, the optimal treatment schema remains uncertain due to data heterogeneity among published studies. Most studies assessing TNT provided patients with fluorouracil- and oxaliplatin-based chemotherapy (10, 24, 25). In addition, irinotecan-based chemotherapy, including doublet regimen CAPIRI (26, 27) and triplet regimen FOFIRINOX (11), showed good toxicity, tumor response rates, and survival outcomes. Most studies that considered neoadjuvant chemotherapy alone provided patients with CapeOX/FOLFOX, with or without monoclonal antibodies. However, whether all patients are eligible for neoadjuvant chemotherapy alone remains debatable. Good tumor response rates were observed when Deng et al. (16) provided CapeOX alone to patients with low- and intermediate-risk LARC. However, the Japanese N-SOG 03 trial revealed that CapeOX plus bevacizumab was associated with a poorer local recurrence and OS rates in patients with cT4b LARC, indicating that chemotherapy alone might not be suitable for the cT4b population (17). In our study, we used CapeOX or mFOLFOX6 regimens for TNT and prolonged neoadjuvant chemotherapy. All patients had baseline resectable tumors, meaning that no cT4b patients were enrolled. We found that doublet regimen CapeOX/mFOLFOX6 was a safe and effective treatment as induction chemotherapy or pNCT alone, but the pCR rate of TNT patients in our study was lower than triplet regimen FOLFIRINOX reported in PRODIGE 23 trial (25% vs 28%) (11).

Chemotherapy cycles are also important. For postoperative chemotherapy, the IDEA study found that 6-months of adjuvant chemotherapy increased the cost and toxicity without improving survival outcomes when compared with 3-month chemotherapy. However, outcomes associated with neoadjuvant chemotherapy may differ from those of adjuvant chemotherapy. Patients who do not undergo surgery have improved the health status and compliance to chemotherapy. In our study, the pNCT group underwent two to four additional cycles of CapeOX/mFOLFOX6 instead of radiation. A comparison of our results with other published studies that assessed the use of neoadjuvant chemotherapy showed that pNCT failed to improve rates of pCR (16.3% versus 6.5%–21.0% for pNCT versus other neoadjuvant therapies, respectively) (13, 16, 17, 28–30). This difference may be explained by differences in the selection of patients and therapy regimens. To determine whether prolonged chemotherapy improves response rates, further prospective studies are needed.

The arrangement of chemoradiotherapy and chemotherapy regimens was another consideration. There are two major modes of TNT: induction chemotherapy plus chemoradiotherapy and chemoradiotherapy plus consolidation chemotherapy. The CAO/ARO/AIO-12 trial compared induction and consolidation chemotherapy in TNT (31, 32). Findings showed that chemoradiotherapy with consolidation chemotherapy was associated with higher pCR rates than induction chemotherapy (25% vs 17%). This can be explained by the longer interval between chemoradiotherapy and surgery. Nevertheless, improvement to pCR failed to improve survival outcomes according to final results of CAO/ARO/AIO-12. In our study, we applied the induction chemotherapy plus chemoradiotherapy followed with an extra cycle of chemotherapy, and achieved a pCR rate of 25% (23/92). Compared with relevant studies (32), our results showed that the additional chemotherapy could probably improve the pCR rate of induction chemotherapy mode. Moreover, based on our experience, induction chemotherapy is more suitable for a “neoadjuvant chemotherapy plus selective radiation” mode. When the scheme was used, patients underwent chemotherapy before their tumor response status was re-evaluated to determine whether additional radiation was needed.

Another consideration is the interval between the last treatment and surgery. The optimal radiotherapy fractionation and timing to surgery is still undetermined. The Stockholm III trial compared short interval (1 week before surgery) with long interval (4-8 weeks before surgery), and showed a comparable oncological outcome between the two groups (33). In a recent randomized study, Akgun et al. (34) compared outcomes of patients for whom intervals between surgery and chemotherapy were either less or more than 8 weeks. The results showed that patients with an interval of more than 8 weeks had improved disease regression and pCR rates compared to those with a surgery-to-chemotherapy interval of less than 8 weeks. Related systematic reviews also showed that surgical delay may improve pCR rates. However, the delayed surgery was not significantly associated with long-term prognosis. The timing to surgery and the best arrangement of neoadjuvant treatment and surgery worth more investigations.

We are aware that this study has some limitations. First, this was a retrospective study with a limited amount of patients. Although we found difference in RFS between the two groups, the study was likely not powered to detect the difference in terms of the recurrence patterns. Second, the functional outcomes of patients after surgery (sexual dysfunction, urinary dysfunction, etc.) were absent, and we failed to evaluate the functional complications between two groups. Third, all enrolled patients were diagnosed with baseline resectable LARC. The efficacy of pNCT for the conversion of unresectable tumors or lateral lymph node metastases was unable to be evaluated. Finally, since patients of both groups completed all cycles of neoadjuvant treatment, we were unable to compare the compliance differences between the two schemas.

In summary, the results of our study show that TNT and pNCT produce similar 3-year RFS and OS rates in patients with mid-to-upper rectal cancer. TNT was associated with improved tumor responsiveness in all patients and improved 3-year RFS rates in those with low rectal cancer. This result indicates that neoadjuvant chemotherapy without radiation might be an option for patients with mid-to-upper rectal cancer. More randomized controlled studies are needed to determine better schema for neoadjuvant therapy.

## Data availability statement

The original contributions presented in the study are included in the article/**Supplementary Material**. Further inquiries can be directed to the corresponding authors.

## Ethics statement

The studies involving human participants were reviewed and approved by Ethics committee of Shanghai Ruijin Hospital. The patients/participants provided their written informed consent to participate in this study.

## Author contributions

MZ and ZH contributed to conception and design of the study. JM, FD and LZ recruited the patients. XZ, PH and LYZ collected the database and performed the statistical analysis. XZ and PH wrote the first draft of the manuscript. MZ and ZH edited the draft. All authors contributed to manuscript revision, read, and approved the submitted version. All authors contributed to the article and approved the submitted version.

## Funding 

This study was supported by the National Natural Science Foundation of China (No.82072614).

## Conflict of interest

The authors declare that the research was conducted in the absence of any commercial or financial relationships that could be construed as a potential conflict of interest.

## Publisher’s note

All claims expressed in this article are solely those of the authors and do not necessarily represent those of their affiliated organizations, or those of the publisher, the editors and the reviewers. Any product that may be evaluated in this article, or claim that may be made by its manufacturer, is not guaranteed or endorsed by the publisher.
