# Supplementary material for: Prolonged neoadjuvant chemotherapy without radiation versus total neoadjuvant therapy for locally advanced rectal cancer: A propensity score matched study
Source: Front Oncol. 2022 Sep 16;12:953790. doi: 10.3389/fonc.2022.953790 (PMC9523158; doi:10.3389/fonc.2022.953790)
Supplement: Supplementary file 1 [file Table_1.docx]

**Supplementary Table 1．Clinical characteristics and treatments of excluded patients underwent additional treatment**

| **Patient** | | **Sex** | **Original treatment** | | | | **RECIST** | **Reason to excluded** | **ycTNM**  **after pNCT/TNT** | **Additional preoperative treatment** | **Surgery** |
| --- | --- | --- | --- | --- | --- | --- | --- | --- | --- | --- | --- |
| pNCT Group | Patient 1 | Male | CapeOX*6 cycles | | | | PD | CRM+ | T4N1M0 | CRT | R2 Resection |
|  | Patient 2 | Male | CapeOX*8 cycles | | | | SD | CRM+ | T3N0M0 | CRT | R0 Resection |
|  | Patient 3 | Male | CapeOX*6 cycles | | | | PD | CRM+ | T4N1M0 | mFOLFOX6+CRT | R2 Resection |
|  | Patient 4 | Female | CapeOX*6 cycles | | | | SD | Lateral lymph node metastasis | T3N2M0 | FOLFIRI+CRT | No |
|  |  |  |  |  |  |  |  |  |  |  |  |
| TNT Group | Patient 1 | Male | CapeOX+CRT | | | | SD | CRM+ | T3N1M0 | FOLFIRI | R2 Resection |
|  | Patient 2 | Male | mFOLFOX6+CRT | | | | SD | CRM+ | T3N2M0 | FOLFIRI | R2 Resection |

PD: progressive disase; SD: stable disase; CRT: chemoradiotherapy
